# The first newborn patient with SARS-CoV-2 variant B.1.1.7 identified in Viet Nam: treatment and care practices

**DOI:** 10.5365/wpsar.2021.12.2.008

**Published:** 2021-08-16

**Authors:** Dem Van Pham, Hai Hoang, Anh Viet Nguyen, Nam Thanh Nguyen, Ngoc Van Hoang, Ngoc-Anh Thi Hoang

**Affiliations:** a Pediatrics Department, Bach Mai Hospital, Hanoi, Viet Nam.; b University of Medicine and Pharmacy, Viet Nam National University, Hanoi, Viet Nam.; c The General Department of Preventive Medicine, Ministry of Health, Hanoi, Viet Nam.; d National Institute of Health Sciences, Bach Mai Hospital, Hanoi, Viet Nam.

## Abstract

SARS-CoV-2 variant B.1.1.7, first detected in September 2020 in the United Kingdom of Great Britain and Northern Ireland, has spread quickly to many countries around the world. While some publications have described the clinical features of adult patients with the B.1.1.7 variant, little information is available on newborn patients. We report the clinical characteristics, treatment and care practices for a 21-day-old newborn patient who was confirmed to be infected with SARS-CoV-2 variant B.1.1.7 in Viet Nam during contact tracing after her father was confirmed to be infected with SARS-CoV-2. The patient displayed no symptoms of COVID-19 on admission but 3 days later developed diarrhoea, vomiting, a runny nose and a productive cough. These symptoms lasted for 3 days before becoming milder for 1 day and then stopping until discharge. During treatment, the patient received Vietnamese traditional herbal peppermint extracts for cough and digestive probiotics for diarrhoeal symptoms. A saltwater solution (Sterimar 0.9%) was used to clean the patient’s sinuses. The patient was cared for and fed breastmilk by her mother, who was provided with personal protective equipment, including sterilized infant equipment, medical masks and hand sanitizer, during hospitalization. The patient’s mother tested negative for SARS-CoV-2 throughout hospitalization. In conclusion, we found no severely abnormal clinical symptoms in a newborn infected with SARS-CoV-2 variant B.1.1.7 during treatment. Our case suggests that newborn patients with the B.1.1.7 variant can receive exclusive breastmilk feeding if sufficient preventive measures are provided for both mother and child.

Coronavirus disease 2019 (COVID-19), a respiratory disease caused by the novel severe acute respiratory syndrome coronavirus 2 (SARS-CoV-2), was first reported in Wuhan, China, in December 2019. Since its spread worldwide, several mutations have been detected, leading to the emergence of multiple genetic variants. The lineage B.1.1.7, or VUI 202012/01, was first detected in September 2020 and has since been detected in numerous countries around the world. (*1*) This variant was reported to have higher transmissibility than the original strain and thus threatened to further burden already overwhelmed health-care systems. Given the limited resources available for studying the clinical features of adult patients with B.1.1.7, little information has been recorded for other vulnerable patients, including newborns. (*2*, *3*)

The SARS-CoV-2 variant B.1.1.7 index case in Viet Nam was recorded in Tokyo, Japan, in a person who had arrived on a flight from Hanoi in late January 2021. After intensive investigation, a large cluster of COVID-19 was found among employees at an industrial production company in Hai Duong Province, northern Viet Nam, all of whom had epidemiological links to the index case. As of mid-March 2021, more than 300 COVID-19 cases had been confirmed among workers at the company and more than 400 secondary community cases among close contacts. This was the largest detected outbreak in Viet Nam since the beginning of the COVID-19 pandemic, with cases confirmed in 13 cities and provinces. Among the confirmed cases in this outbreak, SARS-CoV-2 variant B.1.1.7 was detected in the youngest COVID-19 case: a 21-day-old newborn. We report here the clinical characteristics, treatment and care practices of this case, which is a reminder that COVID-19 can affect infants and children. The report may be useful for both education and clinical practice in COVID-19 case management. It is reported according to the consensus-building clinical case report guidelines. (*4*)

## Patient information

The patient was a 21-day-old female newborn, the firstborn of her mother and father, in Hai Duong Province, northern Viet Nam. Her birth weight was 3200 g. Before admission, she had received exclusive breastfeeding and had no history of adverse obstetric outcomes.

Her father was an employee at the industrial production company involved in the large cluster in January 2021. On 28 January, all employees were mandatorily tested for SARS-CoV-2, and those with epidemiological links to the index case were quarantined. On 29 January, the patient’s father tested positive for SARS-CoV-2 infection and was immediately transferred to a designated hospital for isolation, where he experienced cough, fever and sore throat 1 day later. His daughter (the patient) and his wife (the patient’s mother) were placed in a designated quarantine facility on 30 January. The last close contact (£2 m for more than 15 minutes) between the father and the patient was on 29 January, and the last close contact between the father and the patient’s mother was on 26 January. The father was the patient’s sole caretaker between 26 and 29 January.

During quarantine, the patient was kept in the same room as her mother. On 2 February, after 4 days in quarantine, the patient was confirmed positive for SARS-CoV-2 and immediately admitted to Hai Duong’s COVID-19 specialized mobile hospital.

## Clinical findings and timeline

On admission, the patient was fully conscious, well fed and displayed no respiratory or digestive symptoms. She had no fever and her respiratory rate was 58 breaths per minute. She weighed 3500 g.

**Figure 1:**
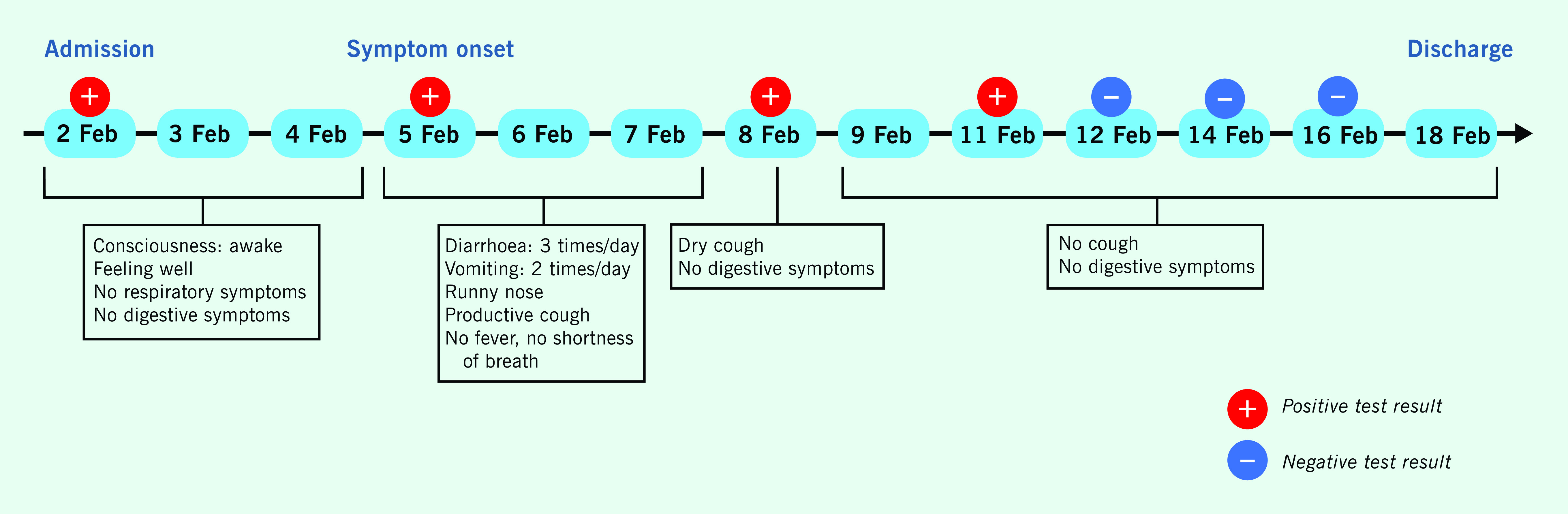
Timeline of the first COVID-19 newborn patient with SARS-CoV-2 variant B.1.1.7, identified in Hai Duong Province, Viet Nam, February 2021

Three days after admission, the patient displayed several symptoms of COVID-19, including diarrhoea, vomiting, runny nose and productive cough. These symptoms lasted for 3 days, during which the frequency of diarrhoea was three times a day and vomiting twice a day. On 8 February, the patient had a dry cough with no digestive symptoms. Between 9 February and discharge, the patient had no cough or other symptoms. The patient was free from fever with no shortness of breath during hospitalization (**Fig. 1**).

[**insert** Figure 1]

The patient’s first positive test for SARS-CoV-2 by reverse-transcription polymerase chain reaction (RT–PCR) was performed on 2 February. The sample was used for genomic sequencing on 3 February for comparison with a sample from the patient’s father: both showed SARS-CoV-2 variant B.1.1.7.

## Therapeutic intervention

The patient was isolated in a negative-pressure room immediately after admission. One paediatrician and two nurses were responsible for her treatment and care. The patient was given a Vietnamese traditional herbal peppermint extract (2 mL, three times per day, 30 minutes before meals) for cough and digestive probiotics (Bio-Flora; 100 mg, once per day) for diarrhoea. Her sinuses were cleaned with a saltwater solution (Sterimar 0.9%; three times per day). These treatments were applied for 5 consecutive days from the date of symptom onset (5 February) to the date of last symptoms (9 February).

The patient was fed eight times per day with breastmilk from her mother in sterilized bottles. The mother was given sterilized breastmilk pumps, hand sanitizer and medical masks throughout, and both she and her infant were monitored closely by health-care staff. The mother was allowed to care for the patient during isolation. She tested negative for SARS-CoV-2 seven times consecutively by RT–PCR.

## Follow-up and outcomes

Hai Duong’s Center for Disease Control and Prevention collected the patient’s oropharyngeal swab samples for SARS-CoV-2 testing by RT–PCR every 3 days during hospitalization and every 2 days from the day of the first negative test, for a total of seven tests during hospitalization (**Fig. 1**). The patient had four positive tests and then three consecutive negative tests for SARS-CoV-2 (48 hours apart, the first negative test on the ninth day of hospitalization) before being discharged on 18 February, for a total of 16 days of hospitalization. During hospitalization, the patient’s vital signs were stable, and the results of all blood tests were in the range of normal values (**Table 1**).

**Table 1:** Clinical testing results for the first COVID-19 newborn patient with SARS-CoV-2 variant B.1.1.7, detected in Hai Duong Province, Viet Nam, February 2021

Test	2 Feb 2021 (Admission)	5 Feb 2021 (Onset)	18 Feb 2021 (Discharge)	Reference range
White blood cells	11.5 × 10^9^/L	8.2 × 10^9^/L	6.2 × 10^9^/L	4–10 × 10^9^/L
Neutrophils	3.1 × 10^9^/L	2.4 × 10^9^/L	3.4 × 10^9^/L	1.9–7.8 × 10^9^/L
Lymphocytes	7.2 × 10^9^/ L	4.8 × 10^9^/L	2.2 × 10^9^/L	0.9–5.2 × 10^9^/L
Platelets	528 × 10^9^/L	311.4 × 10^9^/L	278 × 10^9^/L	140–440 × 10^9^/L
MCHC	123 g/L	107.1 g/L	117.1 g/L	117.1 g/L
Haematocrit	38.9%	30.7%	34.7%	34–487%
Fasting blood sugar	5.5 mmol/L	5.7 mmol/L	5.7 mmol/L	3.6–6.4 mmol/L
C-reative protein	1.2 mg/L	1.5 mg/L	1.5 mg/L	< 5 mg/L
Pro-calcitonin	0.01 mg/L	0.02 mg/L	0.02 mg/L	< 0.05 mg/L
Lactate dehydrogenase	217 U/L	314 U/L	314 U/L	< 450 U/L
Aspartate aminotransferase	23 U/L	28 U/L	28 U/L	< 50 U/L
Alanine aminotransferase	32 U/L	31 U/L	29 U/L	< 50 U/L
Urea	2.8 mmol/L	2.1 mmol/L	3.1 mmol/L	2.8–8.0 mmol/L
Creatinine	24 µmol/L	34 µmol/L	24 µmol/L	< 120 µmol/L
Na^+^	137 mmol/L	136 mmol/L	138 mmol/L	135–145 mmol/L
K+	3.4 mmol/L	3.7 mmol/L	3.5 mmol/L	3.5–5.0 mmol/L
Cl^−^	103 mmol/L	102 mmol/L	101 mmol/L	98–106 mmol/L
Fibrinogen	3.2 g/L	2.8 g/L	3.6 g/L	2.0–4.0 g/L

Thoracic computed tomography was conducted three times and showed no damage to the lung parenchyma.

MCHC: mean corpuscular haemoglobin concentration.

We followed up the infant’s condition after she was discharged and transferred home for a 14-day quarantine. During quarantine, she tested negative for SARS-CoV-2, was well and had no fever and no respiratory or digestive symptoms. She had gained 2500 g since admission to hospital on 2 February.

## Ethics approval

The study protocol was reviewed and the need for approval was waived by the Bach Mai Hospital as a part of routinely conducted disease investigation.

## Discussion

We report the clinical characteristics, treatment and care practices in a 21-day-old newborn in Viet Nam infected with SARS-CoV-2 variant B.1.1.7. The patient was fed her mother’s breastmilk exclusively during hospitalization, both being provided with sufficient preventive measures. The mother tested negative throughout hospitalization.

Clinical data on newborn patients with COVID-19 are still very limited, and most concern mother-to-child transmission of SARS-CoV-2. In the first reported case of neonatal COVID-19 infection in China in February 2020, both the mother and the 3-day-old neonate were confirmed positive. While both patients’ vital signs were stable, the baby displayed no critical clinical features but was not breastfed throughout hospitalization. (*5*) In an observational study of neonates born to COVID-19-infected mothers, one of two newborns tested positive for SARS-CoV-2 at 2 weeks of age while remaining asymptomatic. (*6*) In one study of 10 neonates born to nine mothers with confirmed SARS-CoV-2 infection in China, it was stated that vertical transmission of SARS-CoV-2 could not be confirmed conclusively. No newborns tested positive for SARS-CoV-2 in the first 9 days of life despite adverse symptoms such as fetal distress, premature labour, respiratory distress, thrombocytopenia accompanied by abnormal liver function and even death. (*7*)

Clinical evidence on neonatal COVID-19 infection is also scant in Viet Nam; one 3-month-old infant with SARS-CoV-2 infection, though not the B.1.1.7 variant, was reported in February 2020. Her mild upper respiratory symptoms lasted 6 days, longer than in our patient (4 days). The source of infection was her grandmother, who had tested positive by PCR earlier. The time between the first positive PCR test and the first negative test was shorter than for our patient (7 days versus 9 days). (*8*)

We report the first case of neonatal COVID-19 infection with the B.1.1.7 variant in Viet Nam. The father was infected and was shown to be the source of infection by epidemiological investigation and genetic sequencing. He had strong epidemiological links to a known COVID-19 cluster in the region, while the mother remained uninfected with SARS-CoV-2 throughout the period. The patient was cared for mainly by the father during his infectious period (26–29 January), just before symptom onset on 30 January.

Breastfeeding is a cornerstone of infant development. The World Health Organization recommends that every child be breastfed for at least the first 6 months. (*9*) For infants or mothers infected with SARS-CoV-2, however, concern has been raised about potential vertical transmission through breastfeeding. While there are insufficient data on such a risk, (*10*) there are no firm recommendations for safe breastfeeding of infants with COVID-19. (*11*) In our study, the infant was exclusively fed with breastmilk from sterilized bottles because, at the time, we did not have enough evidence about the risk of transmitting SARS-CoV-2 through direct breastfeeding. Furthermore, no specific process was available for sterilizing the mother’s breasts after breastfeeding, while sterilized breast-pumping equipment and guidance on pumping techniques were accessible and feasible. A previous report from Viet Nam indicated that exclusive breastfeeding of infants with COVID-19 may be safe for the mother, as the infant’s mother did not test positive for SARS-CoV-2 during the infant’s infectious period. (*8*) We considered this practice safe for the mother and the infected child if both adhered to strict infection prevention and control measures. In our case, the mother wore personal protective clothing and an N95 mask during all close contact with the patient in the isolation room, while the patient wore infant masks. Thus, infection prevention and control measures during COVID-19 case management and treatment are very important, especially for infants, for whom constant care and special diets are required.

We acknowledge several limitations to our study. First, although we report the first detected case of a newborn with B.1.1.7 infection in Viet Nam, the findings might not be applicable to a larger population. Given the ever-changing landscape of the COVID-19 pandemic, further studies with larger sample sizes should be conducted to clarify clinical treatment of COVID-19-infected infants. Second, because of the limitations of case reporting, we did not include a control newborn who was uninfected with SARS-CoV-2 or infected with SARS-CoV-2 but not the B1.1.7 variant. Such a comparison would provide important information on the clinical manifestations and appropriate treatment of different mutations of SARS-CoV-2 viruses in infant patients. Third, though imaging investigation was done, the results were not available for our case, although this would have provided insights for clinical practice.

Our study showed no severe abnormal clinical features in a newborn infected with the B.1.1.7 variant. Close monitoring and strict preventive measures are necessary to reduce the risk of cross-transmission between caretakers and patients. Our study suggests that newborn patients with B.1.1.7 can receive exclusive breastfeeding if sufficient personal preventive measures are provided for both mother and child.

DVP was involved in the management of the patient and contributed to the study design, data collection, data interpretation and drafting of the article. HHD, AVN, NVH and NTN performed examinations and data analysis and reviewed the final article. NATH and NVH were involved in drafting and critically revising the article. All authors read and approved the final article.

## Informed consent

Oral informed assent was obtained from the patient’s family for publication of this report.
